# Linear pathway analysis of European botulism poisoning response guidelines

**DOI:** 10.1093/eurpub/ckac131.388

**Published:** 2022-10-25

**Authors:** T Learoyd

**Affiliations:** MCM Medical Affairs, Emergent Biosolutions, Dublin, Ireland

## Abstract

**Background:**

Botulism is a rare illness caused by Clostridium botulinum toxin with a naïve case fatality ratio of 40-50%. There is no coordinated collective worldwide reporting on cases and comparatively few recommendations on case management. This study examined 14 European botulism treatment guidelines.

**Methods:**

A ten-language search was conducted to examine European botulism guidelines. The guidelines were classified by differential diagnosis advice; expert advice access; mention of causalities; contract tracing; biological sampling method; and treatment access rapidity. The guidelines were linearly represented on a probability pathway. Quantified probabilities were entered into the algorithm. Probabilities for algorithmic delay or deviance were estimated or mathematically modeled against Hamiltonian, Ford- Fulkerson and Kruskal pathways. Case recognition was deemed proportional to the availability of information at point of care and produced a hazard function related to a Bayes’ probability model.

**Results:**

Two guidelines did not display all diagnostic information in one place, and six European nations had incomplete descriptions of the chain of causality linking botulism cases: factorially reducing the Borel algorithmic likelihood of diagnosis through contact tracing and decreasing the affectable survival chance. Another limitation was specialist advice and treatment availability in a 48-hour window. Survival probability models to the quoted naïve minimum constraint of a 60% survival factor were depicted, with pharmacokinetic tendential to an exponential decay model. This highlighted the importance of well-constructed case management and logistical stockpiling methods.

**Conclusions:**

In botulism poisoning the 48-hour window is cited as crucial to patient survival chances, to this extent, the availability of clear diagnostic criteria including causation considerations, expert advice access and logistically considered therapeutic stockpiles could improve survival probability.

**Key messages:**

• An international standard for botulism guidance may further improve botulism case identification and survival rates.

• National botulism guidelines with direct contact method to an expert and with strategic positioning of therapeutic stockpiles may reduce time to treatment and improve survival chances.

